# Supplementary material for: Genetic variation at transcription factor binding sites largely explains phenotypic heritability in maize
Source: Nat Genet. 2025 Aug 11;57(9):2313–22. doi: 10.1038/s41588-025-02246-7 (PMC12425805; doi:10.1038/s41588-025-02246-7)
Supplement: Supplementary file 1 — Supplementary Notes 1–8, Figs. 1–19, and References. [file 41588_2025_2246_MOESM1_ESM.pdf]

nature genetics

Supplementary information https://doi.org/10.1038/s41588-025-02246-7

Genetic variation at transcription factor binding sites largely explains phenotypic heritability in maize

In the format provided by the authors and unedited

Table of contents

Supplementary Notes **2**
Supplementary Note 1 2
Supplementary Note 2 3
Supplementary Note 3 3
Supplementary Note 4 5
Supplementary Note 5 5
Supplementary Note 6 6
Supplementary Note 7 7
Supplementary Note 8 8
Supplementary Figures **9**
Supplementary Figure 1 9
Supplementary Figure 2 10
Supplementary Figure 3 11
Supplementary Figure 4 11
Supplementary Figure 5 12
Supplementary Figure 6 12
Supplementary Figure 7 13
Supplementary Figure 8 14
Supplementary Figure 9 14
Supplementary Figure 10 15
Supplementary Figure 11 16
Supplementary Figure 12 17
Supplementary Figure 13 17
Supplementary Figure 14 18
Supplementary Figure 15 19
Supplementary Figure 16 20
Supplementary Figure 17 21
Supplementary Figure 18 21
Supplementary Figure 19 22
Supplementary References **22**

Supplementary Notes

Supplementary Note 1

If haplotype-specific MOA-seq can detect relevant variation in TF binding, we expect it to coincide with potential *cis*-variation in the B73xMo17 F1. We previously reported the allele-specific binding of ZmBZR1 upstream of the TSS of *POLYGALACTURONASE-INHIBITING PROTEIN2* (*ZmPGIP2*, Zm00001eb034870, Extended Data Fig. 1a)[1]. *ZmPGIP2* encodes a cell wall protein and is a candidate locus for both northern and southern leaf blight resistance[2,3]. The overlapping MOA and BZR1 ChIP binding peaks were both significantly higher for the B73 allele compared to Mo17, exhibiting a 5-fold ($p<0.01$) higher MOA coverage (Extended Data Fig. 1a-b). Each of the B73 AMP footprints overlapped with known ZmBZR1 BRRE or G-Box motifs (CGTGTG, CACGTG, and CACGTT, respectively). In contrast, the Mo17 allele contained both a SNP in the BRRE motif as well as a HIP-superfamily helitron insertion between the BRRE and G-Box motifs[1]. In another example, we found a significantly higher (3.21-fold; $p=0.003$) MOA occupancy for the B73 allele for a region upstream of *BARREN INFLORESCENCE2* (*ZmBIF2,* Zm00001eb031760) (Extended Data Fig. 1c-d), along with codirectional changes in transcript abundance (Supplementary Fig. 3). Mutant *bif2* maize plants exhibit reduced formation of tassel branches, spikelets, and ear shoots[4] and natural variation in tassel branch zone length, plant height, and leaf size have been mapped (association mapping validated by linkage mapping in the intermated B73 x Mo17 population) to a small 80 bp hypervariable region in the *ZmBIF2* promoter along with allele-specific transcript levels in the B73xMo17 hybrid[5]. Consistent with these previous findings, the MOA-seq AMP footprint at *ZmBIF2* overlaps with the hypervariable region (in Extended Data Fig. 1d).

Supplementary Note 2

Relative water content and soil capacity measurements

At harvest, a 3 cm leaf sheath section below the third leaf ligule was removed for each plant. Four sheath sections (one of each plant in the pot) were processed together to yield one relative water content (RWC) value per pot. First, the samples' fresh weight (FW) was determined, followed by the turgor weight (TW) after 24 h in water at 4 ℃. The dry weight (DW) was measured after several days at 60 ℃ and the RWC was calculated as ((FW-DW)/(TW-DW))*100. Field capacity was measured using a FOM2 Field Operating Meter (ETest, Poland).

Supplementary Note 3

MOA-seq and RNA-seq sample preparation and sequencing

MOA-seq was performed following the procedure described in ref.[6] with minor modifications. Immediately before use, 627 µl 16 % Paraformaldehyde was mixed with 10 ml fixation solution ((for 10 ml: 1.2 ml Buffer A (150 mM PIPES, 800 mM KCl, 200 mM NaCl, 20 mM EDTA, 5 mM EGTA, pH 6.8); 2.4 ml 1,6 M sorbitol; 12 µl 1M DTT; 12 µL 1000x polyamines (0,15 M spermine, 0,5 M spermidine); 10 µl 200 mM phenanthroline; and 20 µl 200 mM PMSF. This mixture was added to 1 g of frozen, finely ground leaf blade tissue. Fixation was performed under constant mixing (40 rpm on a rocker shaker) for 10 minutes, then quenched with 0.25 M glycine for 5 minutes while continuously mixing. The sample was diluted to 45 ml in ice-cold MDB buffer (50 mM HEPES, 12,5% glycerol, 25 mM KCl, 4 mM $MgCl_2$, 1 mM $CaCl_2$, pH 7.6), centrifuged (1500 rcf/10 minutes, 4℃), and the pellet was resuspended in 5 ml MBD with 1% Triton X-100. After adding 20 µl of 200 mM PMSF, the nuclei were released via mechanical disruption using a polytron at 25,000 min^{-1}, followed by 29,000 min^{-1}, three times, each time 10 seconds, all carried out at 4℃. The lysate was filtered through miracloth, washed with MDB buffer, and centrifuged twice (2000 rcf/ 15 minutes, 4℃) to wash the nuclei. Nuclei were resuspended in 550 µl to 1100 µl MBD with 1% Triton X-100 (volume was adjusted to obtain similar density of well-watered and drought-stressed nuclei). Seven

70 µl aliquots were digested with MNase. 7.8 µl of 6.25 U/ml or 12.5 U/ml MNase stock (depending on nuclei concentration) was added into each aliquot tube with quick mixing and incubated at 37℃ for 15 minutes. The reaction was stopped with 0.5 M EGTA-NaOH (pH 8.0) containing 2.5 µl RNAse A (20 mg/ml) for 30 min at room temperature, followed by decrosslinking in a solution with 0.5 M NaCl, 1% SDS, and 0.2 mg/ml Proteinase K at 65℃ for 10 hours. DNA purification included extraction with Chloroform:Isoamylalcohol (24:1), followed by using the NEB Monarch PCR and DNA cleanup kit following the oligonucleotide cleanup instructions. DNA was quantified using Qubit dsDNA HS assay kit (Thermo Fisher Scientific). The library was prepared using the NEBNext® Ultra™ II DNA Library Prep Kit for Illumina®, following the manufacturer's protocol with minor modifications. Ligation was extended to 30 minutes at 20°C. Pre-PCR dual size selection was performed using purification beads (NEBNext®) first with 50 µl (supernatant retained) followed by 100 µl (beads retained). PCR amplification was conducted with five cycles, followed by cleanup with 75 µl purification beads. Eluted DNA was size-selected via BluePippin in a 3% dye-free agarose gel and Q3 marker mix, targeting 195 bp within a 150–240 bp range. Final cleanup was performed using 75 µl purification beads eluted in 15 µl 0.1 TE buffer. Libraries (in pools of 30) were sequenced on Illumina NovaSeq 6000 S4 lanes.

RNA-seq was performed as described in ref.[1]: total RNA was extracted from around 100 mg of frozen tissue using the RNeasy Mini Kit from QIAGEN according to manufacturer's instructions, including DNAseI digestion. RNA-seq libraries were generated with NEBNext ® Ultra™ II Directional RNA Library Prep Kit (Cat # E7760) and NEBNext Poly(A) mRNA Magnetic Isolation Module (Cat # E7490) starting with 5 µg total RNA and minor modifications of the manufacturer's protocol. The fragmentation step was performed at 94°C for seven minutes and a 1:5 dilution of the adaptor was used for ligation. For prePCR dual size selection, we used 26.5 µl (instead of 25 µl) purification beads to be added to the 96.5 µl ligation reaction. The PCR enrichment was performed with 9 cycles and then purified with 35 µl of purification beads. The libraries were sequenced on the Illumina NovaSeq 6000 S4 platform.

Supplementary Note 4

Personalized A619 genome

During this study, the maize inbred A619 had no reference assembly. Of the inbred maize lines with high quality assemblies, A619 is most closely related to Oh43[7]. We downloaded approximately 30x coverage of A619 WGS data from NCBI SRA (SRR8997919, SRR10127976, SRR8907067, SRR5725670, SRR5663982, and SRR5663981) and then mapped it to the Oh43 genome using bwa-mem2 (v2.2.1) [8] Only uniquely mapping reads (q30) were retained. The GATK v4.3.0.0 pipeline in combination with the “best practices workflow” (https://gatk.broadinstitute.org/hc/en-us/sections/360007226651) was used to identify initial A619/Oh43 SNPs and INDELs with the hard filtering settings: “QD < 2.0”, "QUAL < 100.0", "SOR > 3.0", "FS > 60.0", and "MQ < 40.0". We used these initial variants for base- and variant-recalibration, followed by two additional rounds of training based on convolutional neural networks (CNN) to optimize filtration settings (training input setting: “prior=15.0" and “tranche=99”) and, ultimately selected a refined set of biallelic, homozygous A619/Oh43 SNPs and INDELs. G2Gtools (v. 0.2.7, https://churchill-lab.github.io/g2gtools/) was employed to integrate SNPs and INDELs into the Oh43 genome and generate a A619 pseudo-reference fasta and gff3 file. The average SNP mismatch rate of respective MOA reads from the F1 to the B73xA619 genome was 0.1%, only twice that of the average high-quality B73xNAM genomes (0.04-0.05%), and substantially lower than mapping to either B73 or the A619 psuedo-reference alone (~0.54%).

Supplementary Note 5

MOA-seq whole genome sequencing control

To avoid potential quantification artifacts due to artificial deviation from the expected 1:1 allele ratio (e.g., read mapping errors), we used equal amounts of whole genome sequencing reads of each haplotype treated and analyzed like MOA reads as control. WGS

reads for all lines were downloaded from NCBI SRA[9–12]. All reads were quality trimmed using seqtk (https://github.com/lh3/seqtk) trimfq (v1.3 r106), then cropped to 65 bp length using trimmomatic (v0.39)[13] and 600 million reads per haplotype (except B73xIL14H with 300 million reads each) were extracted at random using seqtk sample (seed -s 100). Per F1, B73 and parental reads were merged and mapped as single-end reads to the F1 genome using STAR (2.7.7a) with the same parameters as described for MOA reads in the main methods section. Bam files were converted to bigwig/bedgraph using bamCoverage with effective genome size determined for MOA reads as described above.

Supplementary Note 6

Isolation and transformation of protoplasts

Isolation and transformation of protoplasts was performed as described in ref.[14]: B73 seeds were pre-germinated by soaking the seeds in demineralized water overnight at room temperature, followed by two days on wet paper towels. The seeds were then planted in soil (1:1 Jongkind soil nr3: sowing soil nr1) and grown at 26℃ in darkness for 10-11 days. The middle third of leaf blades of the second leaf were stacked in piles of 6 leaves and finely chopped using a razor blade. 12 leaves were used to obtain ~ 5 ml of 1x 10^6 protoplasts/ml. Chopped leaves placed in a 250ml-Erlenmeyer flask were vacuum infiltrated with 10 ml protoplast buffer (0.585 M Mannitol, 10 mM KCl, 10 mM MES, pH 5.7 with 6 mg/ml cellulase (Duchefa Biochemie, C8003.0010) and 1.4 mg/ml macerozyme (Duchefa Biochemie, M8002.0010) gently dispersed with a spatula; before use the buffer was incubated at 55℃ for 5 min, while swirling the tube, followed by 3 min on ice, then 1mM CaCl2 and 0.02% BSA were added) for 40 min in darkness (flask covered in aluminium foil). In the beginning of the procedure, the vacuum was applied and released 10 times before the 40 min incubation. Flasks were placed at 26℃ in the dark under gentle shaking at 60 rpm for 3h, followed by 5 min of shaking at 90 rpm to facilitate protoplast release from the tissue. Protoplasts were filtered through four layers of 50 µm nylon mesh and centrifuged for 2 min at 500 g, 4℃ with

slow acceleration and braking. The supernatant was aspirated through one 1 ml plus one 200 µl filter tip using a vacuum device (leaving ~1 ml) and protoplasts were resuspended in 10 ml protoplast buffer by gentle swirling (10 ml of buffer used to wash out the remaining protoplasts from the flask were filtered through the same nylon mesh and added to the tube). After renewed centrifugation and removal of the supernatant, protoplasts were washed with 15 ml fresh protoplast buffer, and after another centrifugation resuspended in 2-3 ml of fresh protoplast buffer. The protoplast concentration was determined using a counting chamber and the protoplasts were diluted to ~ 1x 10^6 protoplasts/ml and stored on ice until transformation.

For transformation via electroporation, 150 µl of protoplast were added to 150 µl of protoplast buffer containing 10-20 µg purified (NucleoBond Xtra Midi, Macherey-nagel, cat nr MN 740410.50) plasmid DNA in an electroporation cuvette (2 mm; BTX15447270) on ice and mixed by gently pipetting up and down five times. Electroporation was performed at 105 V, 5 msec pulse (two pulses), and 950 msec interval (BTX ECM830 device). Protoplasts were subsequently kept on ice for 10 min and resuspended in 700 µl ice-cold protoplast buffer, transferred to 1.5ml tube tube and then stored in the dark at 20°C for 18-22 h in a tilted position.

Supplementary Note 7

Dual Luciferase assay

Dual Luciferase assays were performed as described in ref.[14]: Transformed maize protoplasts were sedimented by centrifugation at 250 g for 2 min at room temperature. After removal of the supernatant, protoplasts were lysed in 80 µl 1x Passive Lysis Buffer (diluted 5x Passive Lysis Buffer, Promega, Cat. Nr. E194A) followed by vigorous shaking for 10 min. Cellular debris was sedimented by centrifuging at 12,000 g for 10 min at 4°C, and 65 µl of supernatant was recovered for further processing. 20 µl of lysed cell extract per sample was loaded in a 96-well plate (Corning Cat. Nr. 3912) followed by Luc activity and renilla luciferase measurements using a GloMax® Navigator (Promega): First automatically 50 µL

Firefly-Luciferase Assay Reagent (FF-LAR) (200 mM Tris-HCl pH 8.0, 15 mM $MgSO_4$, 0.1 mM EDTA pH 8.0, 25 mM DL-1,4-Dithiothreitol (Fisher Scientific), 1mM ATP pH 7.0 (Sigma Aldrich A2383), 0.2 mM Coenzyme A (Sigma Aldrich C3144), 200 µM D-Luciferin pH 6.0-7.0 (Biosynth L8200)) was added to each well and the luminescence was measured. Then automatically 50 µL Renilla-Luciferase Assay Reagent (R-LAR) (25 mM $Na_4P_2O_7$, 10 mM NaAc, 15 mM EDTA, 500 mM Na_2SO_4, 500 mM NaCl, 50 µM phenylbenzothiazole (Santa Cruz Biotechnology, sc-391075), 4 µM h-coelenterazine (Nanolight 301-500)) was added and the luminescence emitted were measured. After the injection of each reagent, incubation times were 2 seconds, followed by 10 seconds of signal integration.

Supplementary Note 8

Analysis of DNA methylation in non-NAM lines

DNA-methylation reads were processed as described in ref.[1] : Quality and adapter trimming was performed using Trimmomatic (version 0.39)[13] and reads were uniquely mapped to the respective genomes (Mo17 CAU v1, W22 v2 [10], A188 v1 [11]) using Bismark (v0.22.3)[15] with bowtie2 (2.4.4)[16]. Methylation counts were obtained using the bismark methylation extractor with -CX option. Awk was used to convert CX reports into bedgraph followed by bedGraphToBigWig (v4)[17] for visualisation.

Supplementary Figures

Supplementary Figure 1

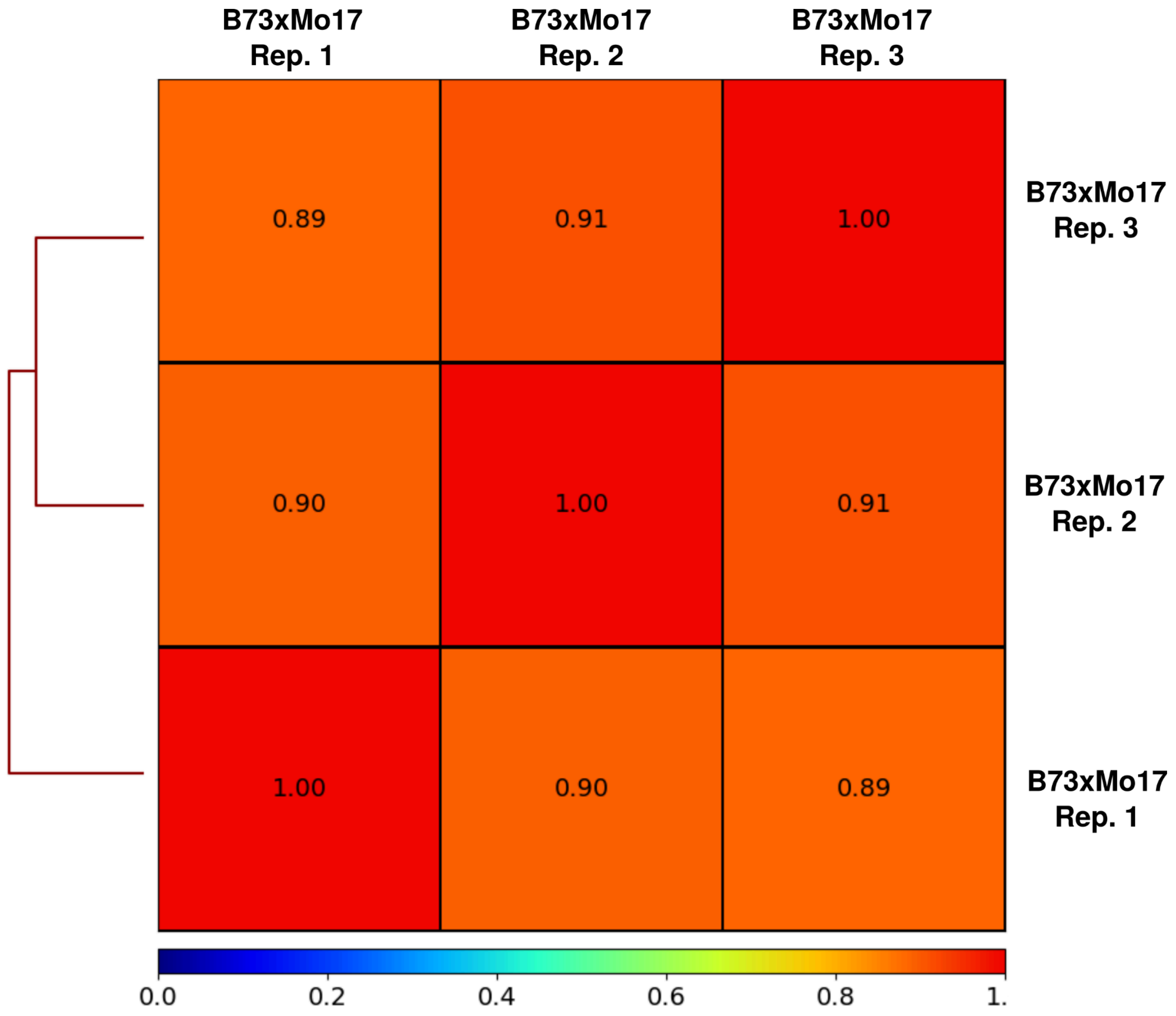

Supplementary Figure 1: **Pearson correlation coefficients for MOA-seq replicates of B73xMo17.**

Supplementary Figure 2

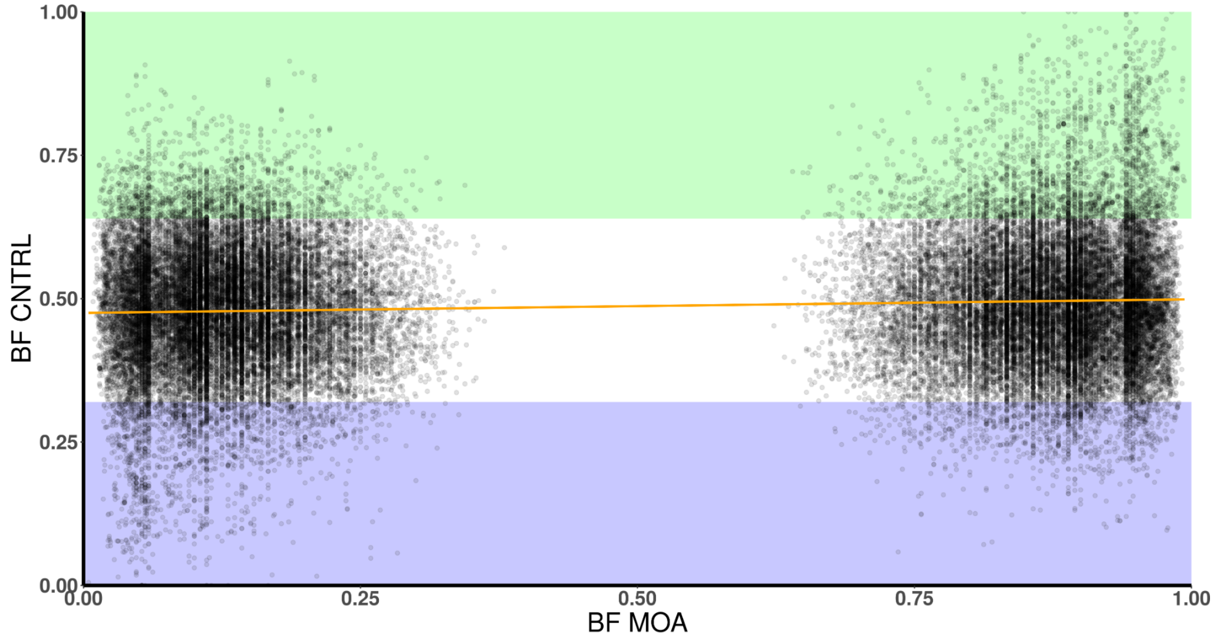

Supplementary Figure 2: Visualization of AMP correction via whole genome sequencing control. Each dot represents one AMP, blue and green shaded areas indicate the fraction of AMPs that were excluded due to bias in counts towards one allele in the control data. The orange line represents a linear regression.

Supplementary Figure 3

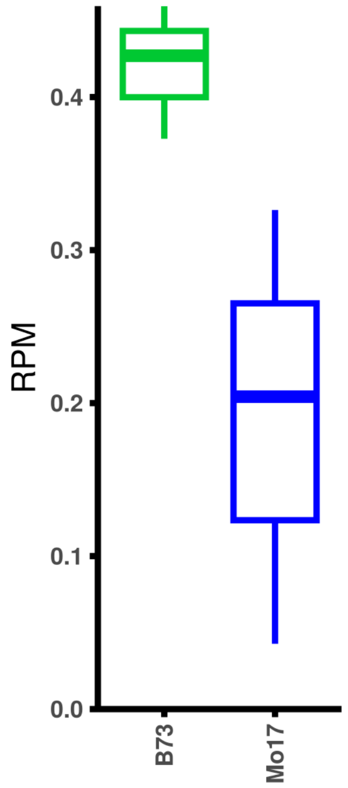

Supplementary Figure 3: **Allele-specific mRNA abundance of *ZmBIF2* measured by RNA-seq in three biological replicates.** Boxes in plots denote the range from the first to the third quartile, lines within boxes indicate the median. Whiskers represent 1.5-fold of the interquartile range.

Supplementary Figure 4

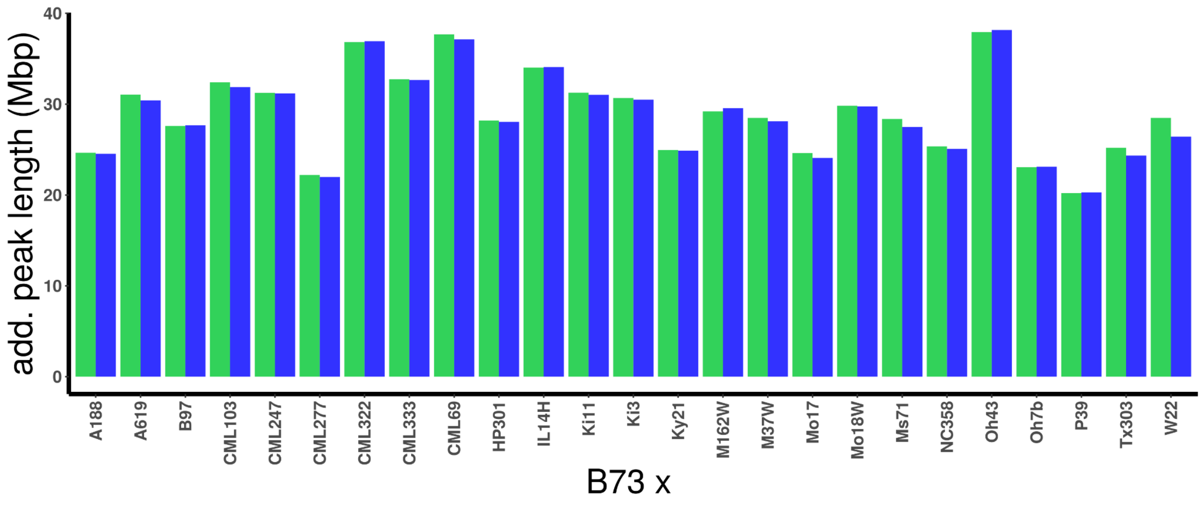

Supplementary Figure S4: **Bases covered by MOA-peaks in each hybrid for the B73 genome fraction (green) and the NAM genome fraction (blue) in Megabases.**

Supplementary Figure 5

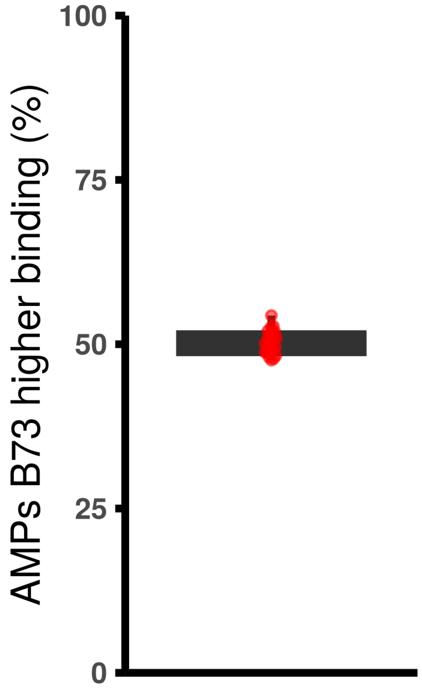

Supplementary Figure 5: **Fraction of AMPs with higher binding towards the B73 allele in the 25 hybrids.** The Box denotes the range from the first to the third quartile, lines within boxes indicate the median. Whiskers represent 1.5-fold of the interquartile range.

Supplementary Figure 6

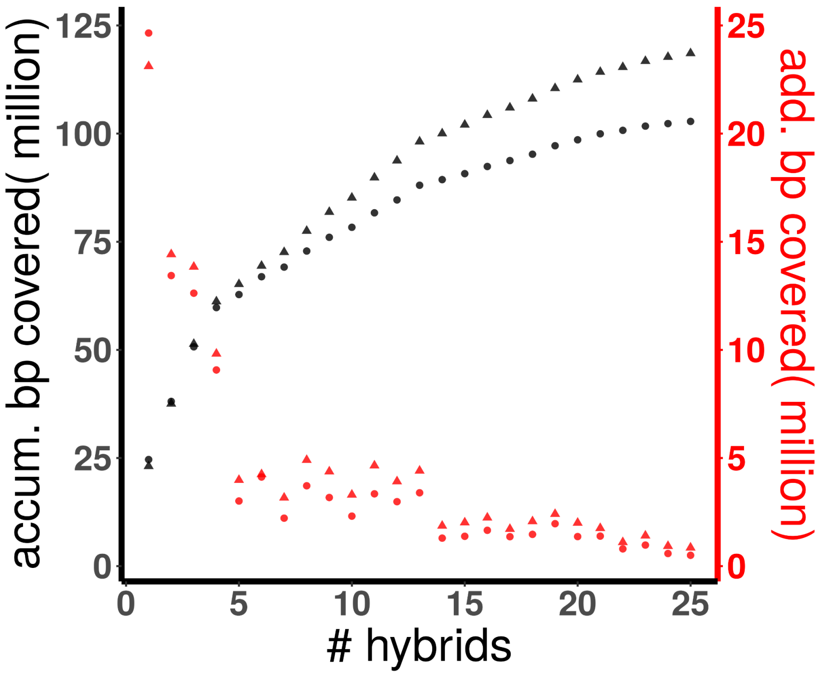

Supplementary Figure S6: **Cumulative number of bp covered by MOA-seq peaks in the 25 F1 hybrids (black) and number of base pairs added by including each hybrid (red).** B73 parts (circles) for each hybrid and NAM parts (triangles) for each hybrid genome are depicted separately. Peak coordinates for the NAM parts of each genome were projected to the B73 genome to allow this comparison.

Supplementary Figure 7

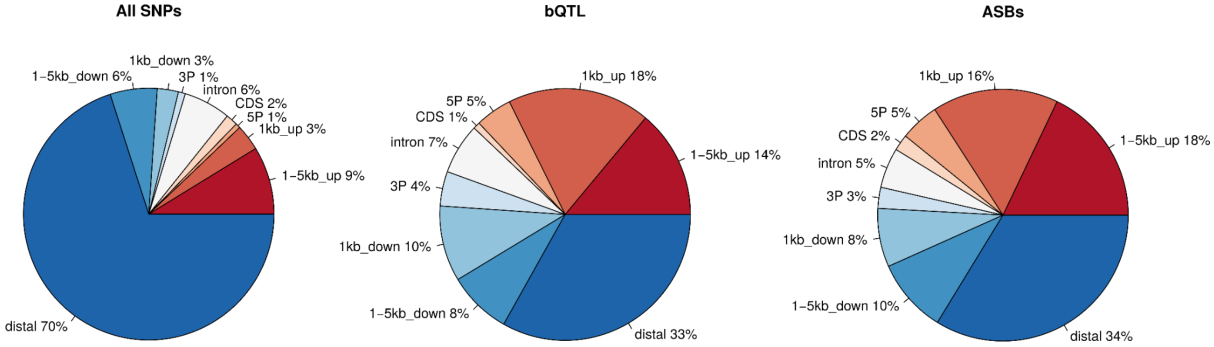

Supplementary Figure 7: **Pie charts displaying the distribution of SNP positions relative to genes.** All SNPs are all SNPs tested in this study, i.e. all biallelic, one-to-one mappable SNPs occurring in at least two lines and bQTL are WW bQTL, ABS are allele-specific binding sites of ZmBZR1[1].

Supplementary Figure 8

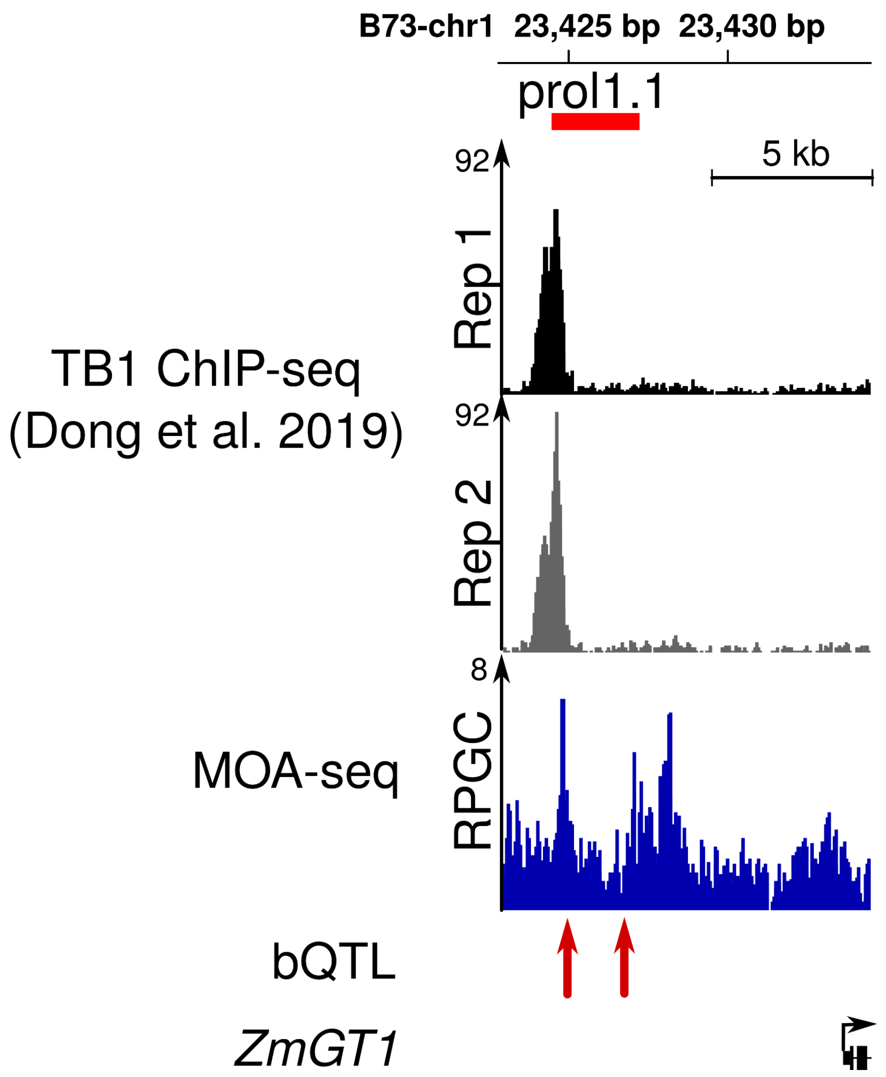

Supplementary Figure 8: **Genome browser view of bQTL region of *ZmGT1* with TB1 binding sites (TB1 binding data from Dong et al. 2019[18]).** bQTL locations are indicated with vertical red arrows. Scale bar applies to all genomic tracks.

Supplementary Figure 9

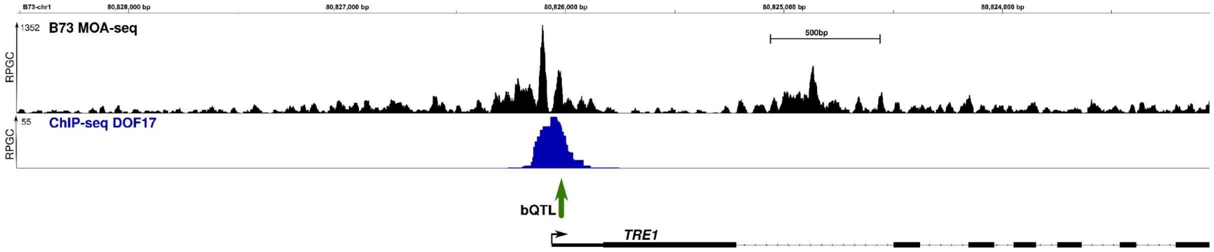

Figure S 9: **Genome browser view of the bQTL (green arrow) in the *ZmTRE1* 5'UTR analyzed in Fig. 3a with B73 MOA high-resolution track and ZmDOF17 binding site[19].** Scale bar applies to all genomic tracks.

Supplementary Figure 10

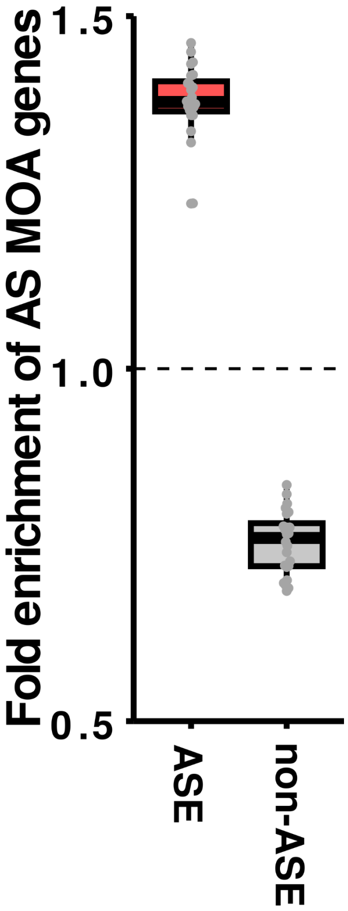

Supplementary Figure 10: **ASE and non-ASE genes under DS conditions are significantly more and less enriched for AMPs in their 3 kb promoter upstream of the TSS, respectively.** Boxes denote the range from the first to the third quartile, lines within boxes indicate the median. Whiskers represent 1.5-fold of the interquartile range.

Supplementary Figure 11

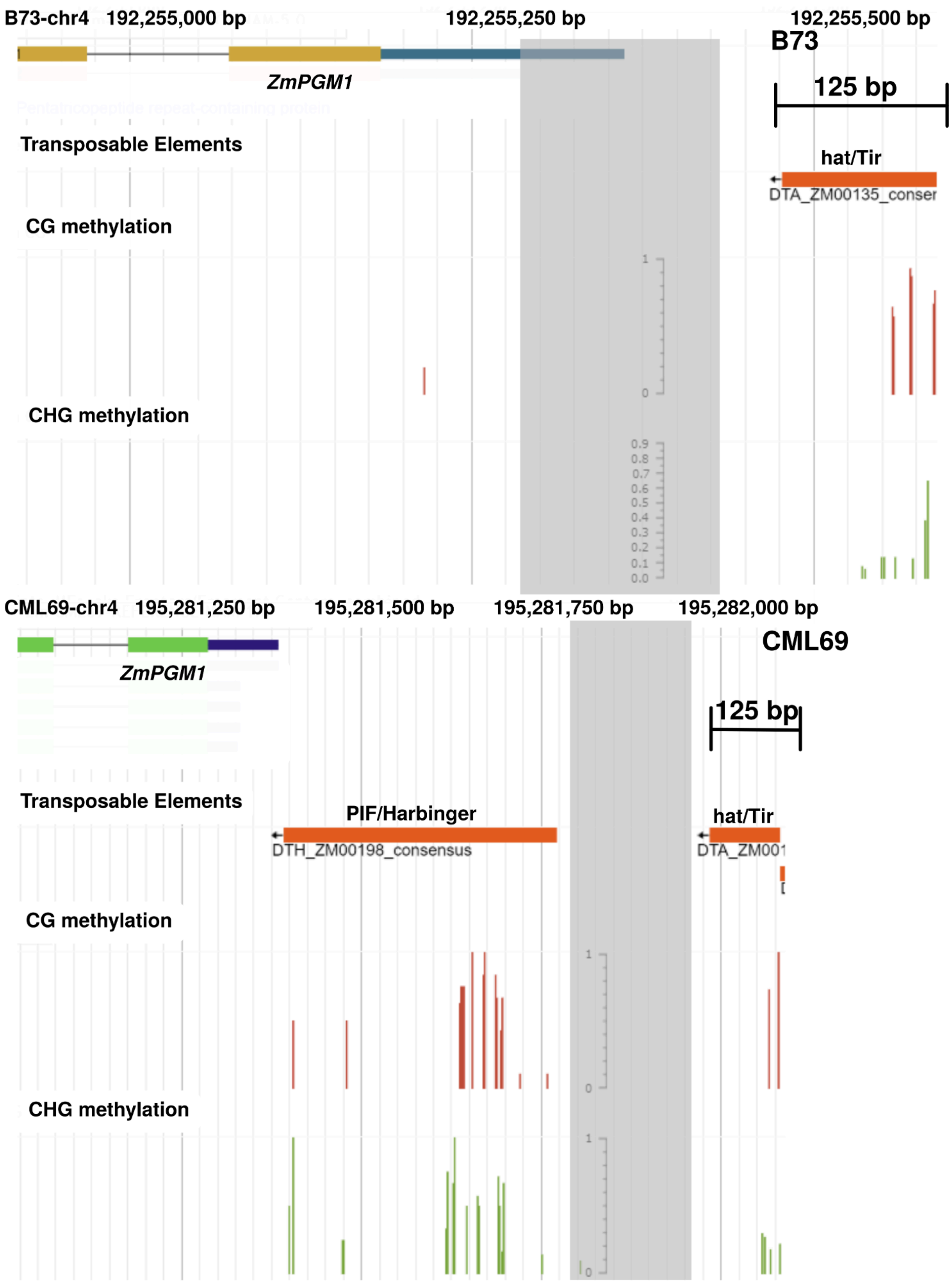

Supplementary Figure 11: **Genome browser view of *ZmPGM1* promoter alleles in B73 and CML69.** The gray bars mark homologous regions in the two genomes where MOA binding is observed in Fig. 3e. A methylated PIF/Harbinger TE is inserted between the gene and the binding region in CML69.

Supplementary Figure 12

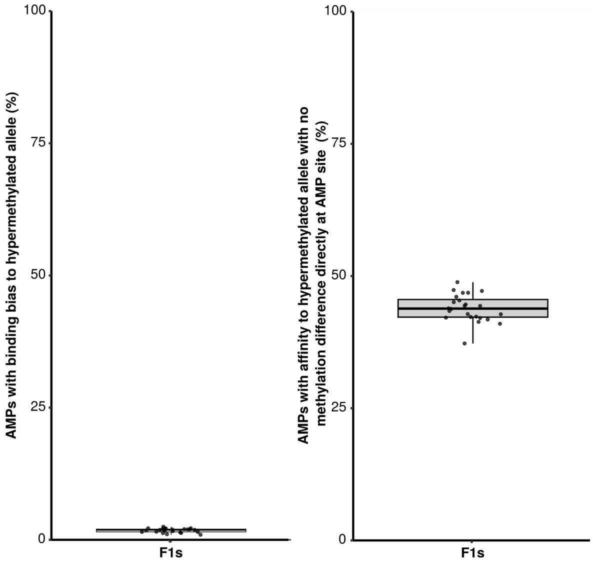

Supplementary Figure 12: **Methylation in 10 bp surrounding bound, hypermethylated AMPs. Left panel: Percentages of AMPs with a stronger binding to the hypermethylated allele for each hybrid.** Right panel: Percentages of hypermethylated (in +-20bp around the AMP), bound AFPs that display no differential methylation in +- 5bp surrounding the AMP. Boxes in plots denote the range from the first to the third quartile, lines within boxes indicate the median. Whiskers represent 1.5-fold of the interquartile range.

Supplementary Figure 13

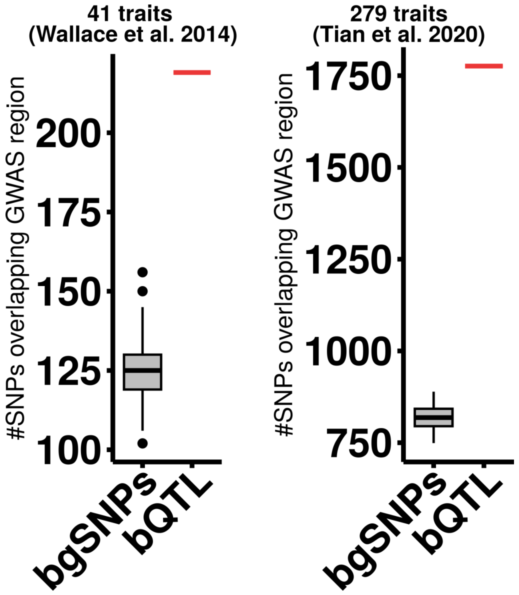

Supplemental Figure 13: **bQTL are enriched in GWAS hits. Enrichment GWAS hits (lead SNP +/- 100 bp) among bQTL compared to 100 bootstrapped sets of matched background SNPs across two curated datasets of 41 and 279 traits[20,21].** Boxes in plots

denote the range from the first to the third quartile, lines within boxes indicate the median. Whiskers represent 1.5-fold of the interquartile range.

Supplementary Figure 14

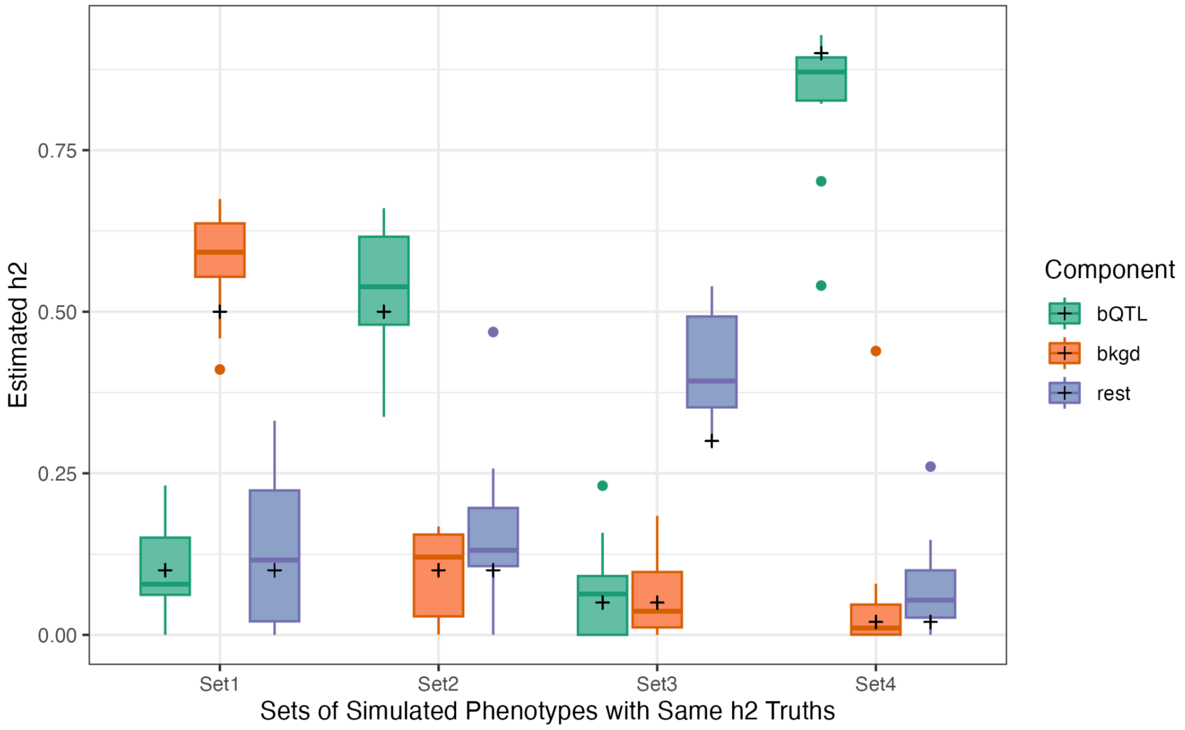

Supplementary Figure 14: **Simulated trait heritabilities.** 10 traits were simulated per 4 sets of heritability scenarios of each component (bQTL, background, rest) and then ran through the VCAP. Estimates for the 10 traits heritability are shown as boxplots, color by component. The black cross represents the known heritability of the traits simulated for a given set. Boxes in plots denote the range from the first to the third quartile, lines within boxes indicate the median. Whiskers represent 1.5-fold of the interquartile range.

Supplementary Figure 15

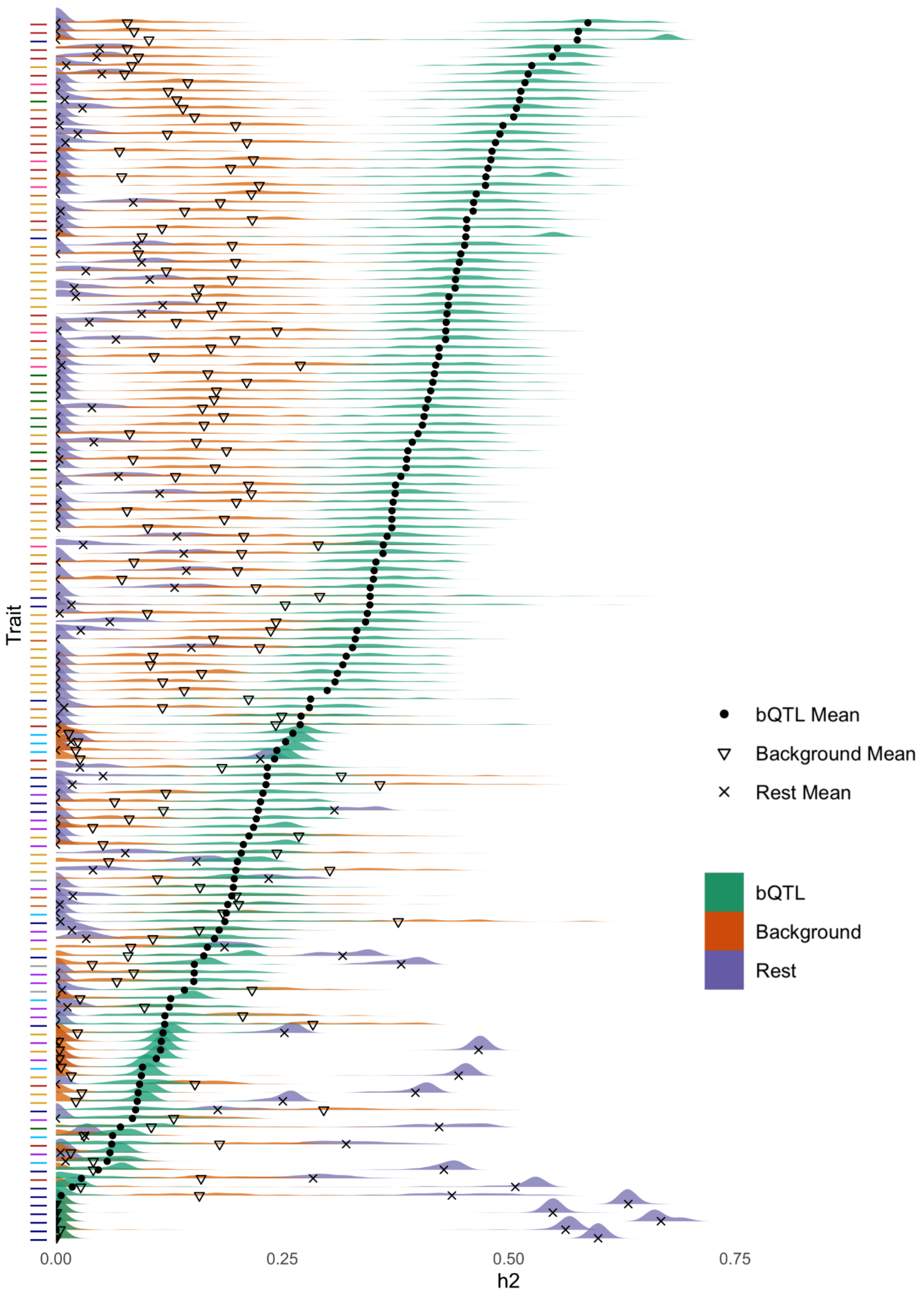

Supplementary Figure 15: **Estimated additive heritability organized by 143 traits.** Colored ridges show the estimated additive genetic variance across 100 permutations for either MOA bQTL (only those purely depending on the genotype) (green), bgSNPs (orange), and rest (purple) components. Black symbols represent the mean estimated value across permutations. Traits arranged by bQTL mean variance estimates and color coded according to general trait groupings: vitamin E metabolites = navy blue, metabolites = purple, stalk strength = light blue, flowering time = gold, plant architecture = red, disease = green, tassel architecture = pink, ear architecture = orange, misc. = gray.

Supplementary Figure 16

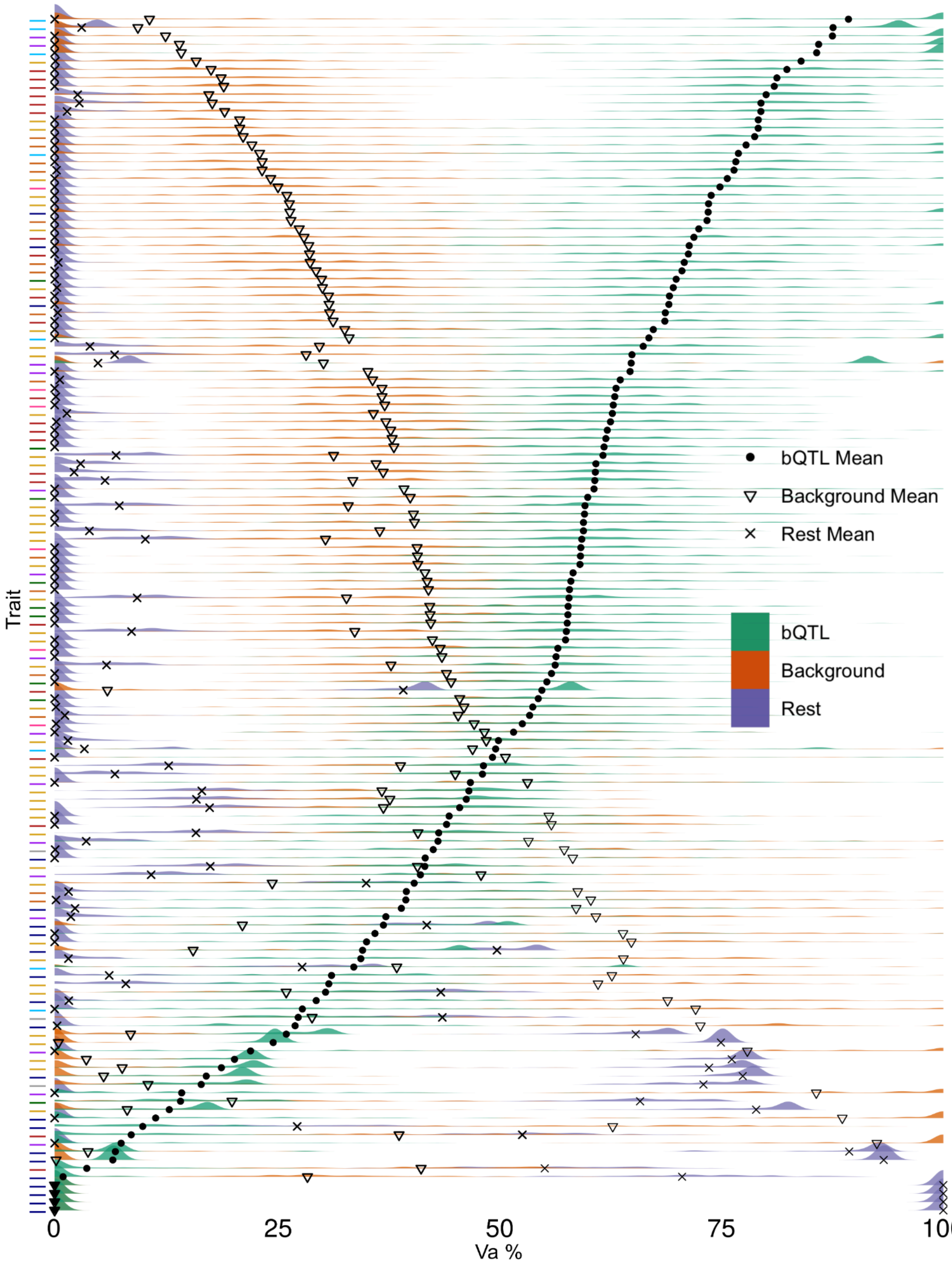

Supplementary Figure 16: **Estimated additive genetic variance for bQTL depending on genotype (including those depending on genotype and methylation) organized by 143 traits.** Colored ridges show the estimated additive genetic variance across 100 permutations for either bQTL depending on genotype or genotype and methylation (green), bgSNPs (orange), and rest (purple) components. Black symbols represent the mean estimated value across permutations. Traits arranged by bQTL mean-variance estimates and color-coded according to general trait groupings: vitamin E metabolites = navy blue, metabolites = purple, stalk strength = light blue, flowering time = gold, plant architecture = red, disease = green, tassel architecture = pink, ear architecture = orange, misc. = gray.

Supplementary Figure 17

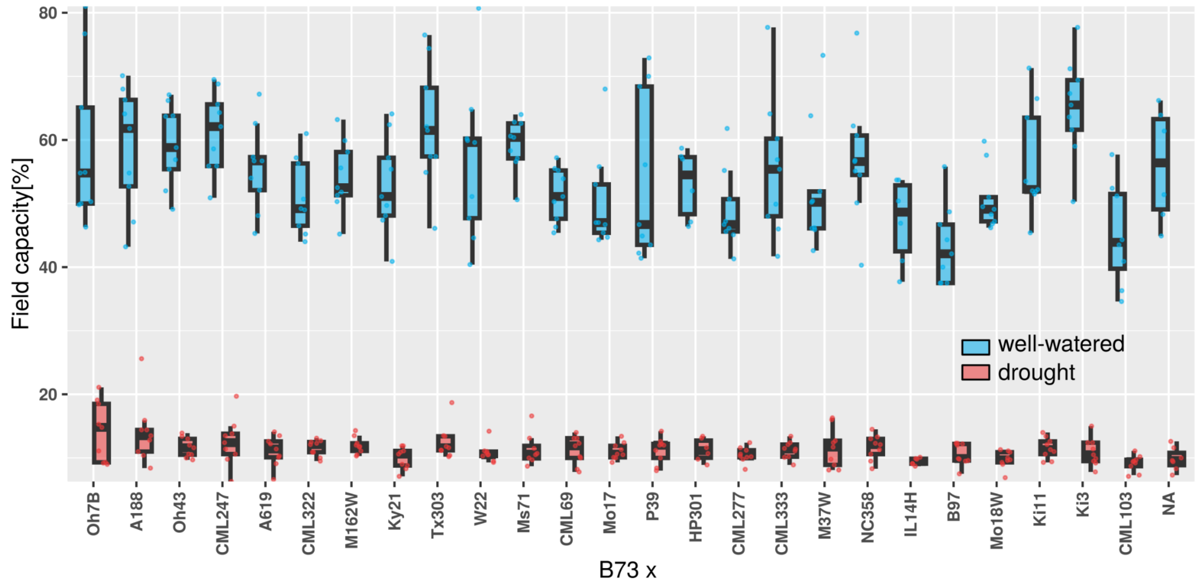

Supplementary Figure 17: **Field capacity in pots at the moment of harvest.** n=9 pots with 4 plants each. Boxes in plots denote the range from the first to the third quartile, lines within boxes indicate the median. Whiskers represent 1.5-fold of the interquartile range.

Supplementary Figure 18

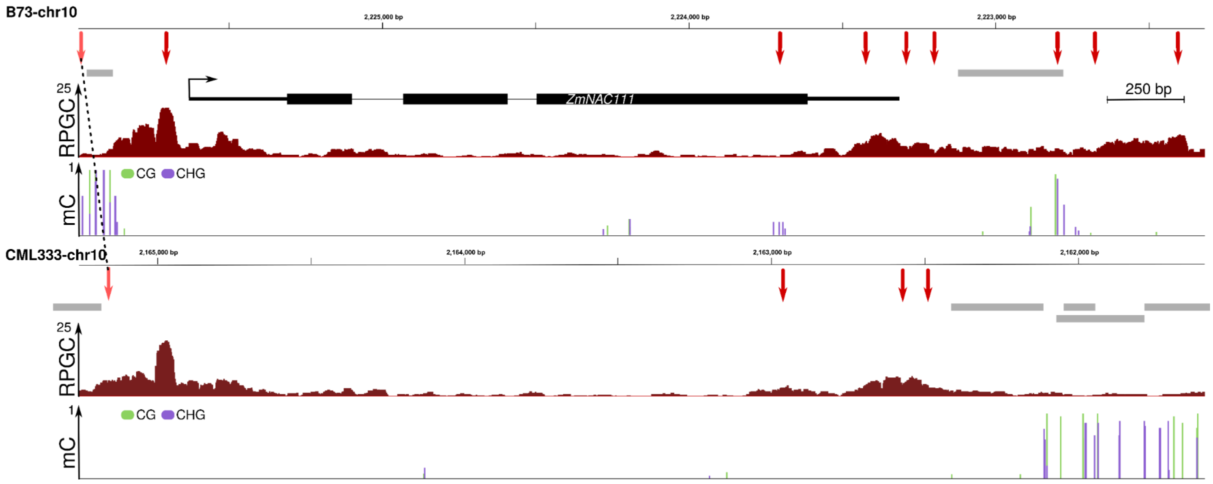

Supplementary Figure 18: **Genome browser view of MOA-seq and DNA methylation levels at the *ZmNAC111* locus under drought conditions.** The promoter region carries a Tc1/mariner TE, previously shown to influence DS response and *ZmNAC111* expression[22] in B73 (upper panel) but not in CML333 (lower panel). The bQTL are indicated by vertical red arrows for both genomes. TEs are indicated as gray bars. The light red marked SNP (leftmost arrow) was just below the threshold for bQTL (with an FDR corrected p-value of 0.07). This region carries a Tc1/mariner TE in B73 but not CML333. Please note that DS-bQTL indicated for B73 mark MOA differences significantly associated with genotype/methylation for the entire 25 F1 population, many of which may not be variants between the example B73 and CML333 shown here. Therefore, the bQTL with variants present in CML333 are indicated above the CML333 track (also red arrows). Scale bar applies to all genomic tracks.

Supplementary Figure 19

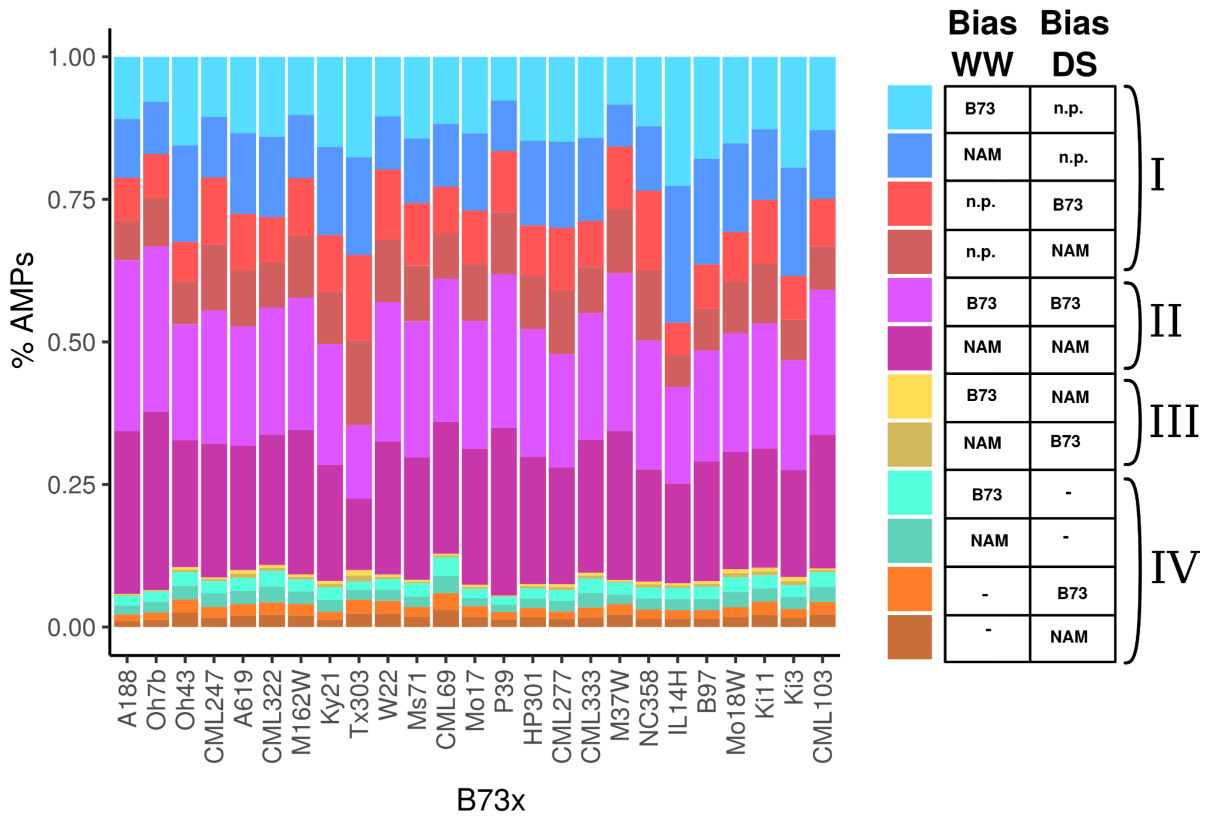

Supplementary Figure 19: **Comparison of haplotype-specific binding between WW and DS.** All AMPs are categorized according to their binding bias in WW and DS. Binding frequencies >= 0.6 and <= 0.4 were considered biased. The Roman numerals indicate the same four categories displayed in Fig. 7d for the Oh43 hybrid - I: Bias in one condition but no binding in the other; II: Bias in the same direction in both conditions; III: Bias in opposite directions; IV: Bias in one condition but no bias in the other condition.

Supplementary References

1. Hartwig, T. *et al.* Hybrid allele-specific ChIP-seq analysis identifies variation in brassinosteroid-responsive transcription factor binding linked to traits in maize. *Genome Biol.* **24**, 108 (2023).
2. Balint-Kurti, P. J. *et al.* Precise mapping of quantitative trait loci for resistance to southern leaf blight, caused by *Cochliobolus heterostrophus* Race O, and flowering time using advanced intercross maize lines. *Genetics* **176**, 645–657 (2007).
3. Omondi, D. O. *et al.* Combination of linkage and association mapping with genomic

prediction to infer QTL regions associated with gray leaf spot and northern corn leaf blight resistance in tropical maize. *Front. Genet.* **14**, (2023).

4. McSteen, P. & Hake, S. *barren inflorescence2* regulates axillary meristem development in the maize inflorescence. *Development* **128**, 2881–2891 (2001).
5. Pressoir, G. *et al.* Natural variation in maize architecture is mediated by allelic differences at the PINOID co-ortholog *barren inflorescence2*. *Plant J.* **58**, 618–628 (2009).
6. Savadel, S. D. *et al.* The native cistrome and sequence motif families of the maize ear. *PLOS Genet.* **17**, e1009689 (2021).
7. Flint-Garcia, S. A. *et al.* Maize association population: a high-resolution platform for quantitative trait locus dissection. *Plant J.* **44**, 1054–1064 (2005).
8. Li, H. & Durbin, R. Fast and accurate short read alignment with Burrows–Wheeler transform. *Bioinformatics* **25**, 1754–1760 (2009).
9. Jiao, Y. *et al.* Improved maize reference genome with single-molecule technologies. *Nature* **546**, 524–527 (2017).
10. Springer, N. M. *et al.* The maize W22 genome provides a foundation for functional genomics and transposon biology. *Nat. Genet.* **50**, 1282–1288 (2018).
11. Lin, G. *et al.* Chromosome-level genome assembly of a regenerable maize inbred line A188. *Genome Biol.* **22**, 175 (2021).
12. Hufford, M. B. *et al.* De novo assembly, annotation, and comparative analysis of 26 diverse maize genomes. *Science* **373**, 655–662 (2021).
13. Bolger, A. M., Lohse, M. & Usadel, B. Trimmomatic: a flexible trimmer for Illumina sequence data. *Bioinformatics* **30**, 2114–2120 (2014).
14. Hampf, M. & Gossen, M. A protocol for combined *Photinus* and *Renilla* luciferase quantification compatible with protein assays. *Anal. Biochem.* **356**, 94–99 (2006).
15. Krueger, F. & Andrews, S. R. Bismark: a flexible aligner and methylation caller for Bisulfite-Seq applications. *Bioinformatics* **27**, 1571–1572 (2011).
16. Langmead, B. & Salzberg, S. L. Fast gapped-read alignment with Bowtie 2. *Nat. Methods* **9**, 357–359 (2012).

17. Kent, W. J., Zweig, A. S., Barber, G., Hinrichs, A. S. & Karolchik, D. BigWig and BigBed: enabling browsing of large distributed datasets. *Bioinformatics* **26**, 2204–2207 (2010).

18. Dong, Z. *et al.* The regulatory landscape of a core maize domestication module controlling bud dormancy and growth repression. *Nat. Commun.* **10**, 3810 (2019).

19. Tu, X. *et al.* Reconstructing the maize leaf regulatory network using ChIP-seq data of 104 transcription factors. *Nat. Commun.* **11**, 5089 (2020).

20. Tian, D. *et al.* GWAS Atlas: a curated resource of genome-wide variant-trait associations in plants and animals. *Nucleic Acids Res.* **48**, D927–D932 (2020).

21. Wallace, J. G. *et al.* Association mapping across numerous traits reveals patterns of functional variation in maize. *PLOS Genet.* **10**, e1004845 (2014).

22. Mao, H. *et al.* A transposable element in a NAC gene is associated with drought tolerance in maize seedlings. *Nat. Commun.* **6**, 8326 (2015).
