# Supplementary material for: Genetic variation at transcription factor binding sites largely explains phenotypic heritability in maize
Source: Nat Genet. 2025 Aug 11;57(9):2313–22. doi: 10.1038/s41588-025-02246-7 (PMC12425805; doi:10.1038/s41588-025-02246-7)
Supplement: Supplementary file 2 — Reporting Summary [file 41588_2025_2246_MOESM2_ESM.pdf]

nature portfolio

Corresponding author(s): Thomas Hartwig

Last updated by author(s): May 7, 2025

# Reporting Summary

Nature Portfolio wishes to improve the reproducibility of the work that we publish. This form provides structure for consistency and transparency in reporting. For further information on Nature Portfolio policies, see our Editorial Policies and the Editorial Policy Checklist.

## Statistics

For all statistical analyses, confirm that the following items are present in the figure legend, table legend, main text, or Methods section.

| n/a | Confirmed | |
|---|---|---|
| ☐ | ☒ | The exact sample size (*n*) for each experimental group/condition, given as a discrete number and unit of measurement |
| ☐ | ☒ | A statement on whether measurements were taken from distinct samples or whether the same sample was measured repeatedly |
| ☐ | ☒ | The statistical test(s) used AND whether they are one- or two-sided<br>*Only common tests should be described solely by name; describe more complex techniques in the Methods section.* |
| ☐ | ☒ | A description of all covariates tested |
| ☐ | ☒ | A description of any assumptions or corrections, such as tests of normality and adjustment for multiple comparisons |
| ☐ | ☒ | A full description of the statistical parameters including central tendency (e.g. means) or other basic estimates (e.g. regression coefficient) AND variation (e.g. standard deviation) or associated estimates of uncertainty (e.g. confidence intervals) |
| ☐ | ☒ | For null hypothesis testing, the test statistic (e.g. *F*, *t*, *r*) with confidence intervals, effect sizes, degrees of freedom and *P* value noted<br>*Give P values as exact values whenever suitable.* |
| ☒ | ☐ | For Bayesian analysis, information on the choice of priors and Markov chain Monte Carlo settings |
| ☒ | ☐ | For hierarchical and complex designs, identification of the appropriate level for tests and full reporting of outcomes |
| ☐ | ☒ | Estimates of effect sizes (e.g. Cohen's *d*, Pearson's *r*), indicating how they were calculated |

*Our web collection on statistics for biologists contains articles on many of the points above.*

## Software and code

Policy information about availability of computer code

**Data collection**

Standard Illumina sequencing/demultiplexing pipeline

**Data analysis**

SeqPurge (v2022-07-15)
NGmerge (v0.3)
STAR (v2.7.7a)
SAMtools (v1.9)
unique-kmers.py (https://github.com/dib-lab/khmer/, commit fb65d21)
GNU Awk (v4.2.1)
Bedtools (v2.29.0 and v2.30.0)
bedGraphToBigWig (v 4)
seqtk trimfq (v1.3 r106)
trimmomatic (v0.39)
DeepTools (v3.5.0 and v.3.5.5)
bwa-mem2 (v2.2.1)
GATK (v4.3.0.0)
G2Gtools (v. 0.2.7)
progressive cactus (v1.0.0 2020-04-19)
hallLiftover (hal-release-v2.1)
MACS3 (v3.0.1)
Anchorwave (v1.2.2)
minimap2 (2.27-r1193)

CrossMap (version 0.6.4 and 0.7.0)
wgatools (version 0.1.0)
Julia (1.8.1)
R (4.1.1, 4.1.2, and 4.4.2)
Bismark (v0.22.3)
bowtie2 (v2.4.4)
LDAK (v5.2)
Tassel (v5)
CrossMap ( v0.6.4)

GitHub Repositories:
https://github.com/Snodgras/MOA_Analysis
https://github.com/corn2code/bQTL
https://github.com/jengelhorn/AS-MOA
https://github.com/jengelhorn/AS-RNAseq
https://github.com/Ako31415/FIND-CIS-analysis

For manuscripts utilizing custom algorithms or software that are central to the research but not yet described in published literature, software must be made available to editors and reviewers. We strongly encourage code deposition in a community repository (e.g. GitHub). See the Nature Portfolio guidelines for submitting code & software for further information.

## Data

Policy information about availability of data

All manuscripts must include a data availability statement. This statement should provide the following information, where applicable:

- Accession codes, unique identifiers, or web links for publicly available datasets
- A description of any restrictions on data availability
- For clinical datasets or third party data, please ensure that the statement adheres to our policy

All MOA-seq and RNA-seq raw data generated for this publication have been deposited at NCBI SRA under accession number PRJNA1101486. MOA coverage tacks and peak files have been deposited on Gene Expression Omnibus under GSE294039 and will also be included in future releases at MAIZEDGB. Coverage and binding frequency data for all bQTL is accessible at a custom browser at: https://www.plabipd.de/ceplas/?config=maize_hartwig_config.json and zendono (https://doi.org/10.5281/zenodo.15177272). For convenience concatenated genomes were also deposited at zenodo (https://doi.org/10.5281/zenodo.15177272).

## Research involving human participants, their data, or biological material

Policy information about studies with human participants or human data. See also policy information about sex, gender (identity/presentation), and sexual orientation and race, ethnicity and racism.

| | |
|---|---|
| Reporting on sex and gender | n/a |
| Reporting on race, ethnicity, or other socially relevant groupings | n/a |
| Population characteristics | n/a |
| Recruitment | n/a |
| Ethics oversight | n/a |

Note that full information on the approval of the study protocol must also be provided in the manuscript.

# Field-specific reporting

Please select the one below that is the best fit for your research. If you are not sure, read the appropriate sections before making your selection.

☒ Life sciences ☐ Behavioural & social sciences ☐ Ecological, evolutionary & environmental sciences

For a reference copy of the document with all sections, see nature.com/documents/nr-reporting-summary-flat.pdf

# Life sciences study design

All studies must disclose on these points even when the disclosure is negative.

| | |
|---|---|
| Sample size | We proposed to construct a first-generation pan-cistrome for maize. To do that, we focused on diverse genotypes with high-quality genome assemblies available at the time of the study, that could be crossed under field conditions in Germany. Our results indicate that 25 F1 lines are sufficient for the association analyses and that our population was near saturation (Fig. 2, Supplementary Fig. 6). Four F1 or inbred plants per pot and 3 pots (12 plants total) per treatment and per replicate were chosen. No statistical methods were used to pre-determine sample sizes but our sample sizes are similar to those reported in previous publications (Hartwig et al., 2023, doi:10.1186/s13059-023-02909-w). |

| | |
|---|---|
| Data exclusions | no data was excluded |
| Replication | All experiments unless otherwise stated in the Method section were performed with three biological replicates. Plants were grown at separate time points for the three biological replicate. All attempts of replication were successful. |
| Randomization | All plants were grown in a randomized block design. At the start of the treatment all plants were re-randomized and grown in a randomized block design during the treatment. Harvest order was randomized per each harvest, except that well-watered and drought samples for each line were harvested consecutively to minimize differences. |
| Blinding | Blinding was not required as there are no participant biases involved in this plant molecular genetics and genomics work. |

# Reporting for specific materials, systems and methods

We require information from authors about some types of materials, experimental systems and methods used in many studies. Here, indicate whether each material, system or method listed is relevant to your study. If you are not sure if a list item applies to your research, read the appropriate section before selecting a response.

## Materials & experimental systems

| n/a | Involved in the study | |
|---|---|---|
| ☒ | ☐ | Antibodies |
| ☒ | ☐ | Eukaryotic cell lines |
| ☒ | ☐ | Palaeontology and archaeology |
| ☒ | ☐ | Animals and other organisms |
| ☒ | ☐ | Clinical data |
| ☒ | ☐ | Dual use research of concern |
| ☐ | ☒ | Plants |

## Methods

| n/a | Involved in the study | |
|---|---|---|
| ☒ | ☐ | ChIP-seq |
| ☒ | ☐ | Flow cytometry |
| ☒ | ☐ | MRI-based neuroimaging |

# Dual use research of concern

Policy information about dual use research of concern

## Hazards

Could the accidental, deliberate or reckless misuse of agents or technologies generated in the work, or the application of information presented in the manuscript, pose a threat to:

| No | Yes | |
|---|---|---|
| ☒ | ☐ | Public health |
| ☒ | ☐ | National security |
| ☒ | ☐ | Crops and/or livestock |
| ☒ | ☐ | Ecosystems |
| ☒ | ☐ | Any other significant area |

## Experiments of concern

Does the work involve any of these experiments of concern:

| No | Yes | |
|---|---|---|
| ☒ | ☐ | Demonstrate how to render a vaccine ineffective |
| ☒ | ☐ | Confer resistance to therapeutically useful antibiotics or antiviral agents |
| ☒ | ☐ | Enhance the virulence of a pathogen or render a nonpathogen virulent |
| ☒ | ☐ | Increase transmissibility of a pathogen |
| ☒ | ☐ | Alter the host range of a pathogen |
| ☒ | ☐ | Enable evasion of diagnostic/detection modalities |
| ☒ | ☐ | Enable the weaponization of a biological agent or toxin |
| ☒ | ☐ | Any other potentially harmful combination of experiments and agents |

## Plants

| | |
|---|---|
| Seed stocks | B73, Mo17, A619, W23, W22, A188, and US-NAM seeds were supplied by the GRIN National Agricultural Library. |
| Novel plant genotypes | n/a |
| Authentication | In addition to GRIN documentation, whole genome alignment was performed and sequence variants validated in the sequiencing data to ensure genotype authenticity |
